# The integration of emerging omics approaches to advance precision medicine: How can regulatory science help?

**DOI:** 10.1017/cts.2018.330

**Published:** 2018-12-06

**Authors:** Joan E. Adamo, Robert V. Bienvenu, F. Owen Fields, Soma Ghosh, Christina M. Jones, Michael Liebman, Mark S. Lowenthal, Scott J. Steele

**Affiliations:** 1 Clinical & Translational Science Institute, University of Rochester Medical Center, Rochester, NY, USA; 2 Department of Anthropology, University of Maryland, College Park, MD, USA; 3 Worldwide Safety and Regulatory Strategy, Pfizer Research and Development, Collegeville, PA, USA; 4 Division of Molecular Genetics and Pathology, Office of In Vitro Diagnostics and Radiological Health, Center for Devices and Radiological Health, Food and Drug Administration, Silver Spring, MD, USA; 5 Chemical Sciences Division, National Institute of Standards and Technology, Gaithersburg, MD, USA; 6 IPQ Analytics, LLC, Kennett Square, PA, USA; 7 Biomolecular Measurement Division, National Institute of Standards and Technology, Gaithersburg, MD, USA; 8 Clinical & Translational Science Institute and Department of Public Health Sciences, University of Rochester Medical Center, Rochester, NY, USA

**Keywords:** Regulatory science, precision medicine, omics, US Food and Drug Administration, Clinical and Translational Science Award

## Abstract

Building on the recent advances in next-generation sequencing, the integration of genomics, proteomics, metabolomics, and other approaches hold tremendous promise for precision medicine. The approval and adoption of these rapidly advancing technologies and methods presents several regulatory science considerations that need to be addressed. To better understand and address these regulatory science issues, a Clinical and Translational Science Award Working Group convened the Regulatory Science to Advance Precision Medicine Forum. The Forum identified an initial set of regulatory science gaps. The final set of key findings and recommendations provided here address issues related to the lack of standardization of complex tests, preclinical issues, establishing clinical validity and utility, pharmacogenomics considerations, and knowledge gaps.

## Introduction

Regulatory science is defined by the US Food and Drug Administration (FDA) as “the science of developing new tools, standards, and approaches to assess the safety, efficacy, quality and performance of publicly regulated products” [1] that ultimately enhances the overall translational research process and improves the development of safe and effective medical interventions. The increased focus on precision medicine holds tremendous promise to utilize genomic (and other omic information), environmental, lifestyle, and other factors to more effectively guide medical decisions and target treatments to those individuals most likely to have a benefit [2,3]. However, there are a number of regulatory science challenges to ultimately develop and utilize personalized medicine technologies and approaches.

A working group under the National Institutes of Health (NIH) Clinical and Translational Science Award (CTSA) network was proposed by the University of Rochester to help identify and address some of the key topics and opportunities for regulatory science to advance precision medicine. The working group identified two topics of focus for the 2017 Regulatory Science to Advance Precision Medicine Forum, where 38 experts further evaluated these key topics to identify regulatory science gaps and specific regulatory considerations, recommend potential approaches to address these regulatory science gaps, and provide suggestions for the development of educational resources (see Appendix 1).

One topic discussed at the 2017 Forum was technologies and approaches that integrate and analyze genomic, proteomic, metabolomic, and/or epigenetic data for precision medicine (an additional topic, *3D Printing of Medical Products*, was also discussed and will be the subject of a partner publication). This communication expands on the initial findings from the group focused on omics and precision medicine, and provides a set of recommendations for each of the key areas identified. The meeting was framed by introducing both the emerging science and regulatory considerations.

## Emerging Science

“Omic” technologies enable comprehensive identification and quantitation of the components that make up a cell, tissue, or organism via assay designs that take advantage of massive multiplexing and parallelism. These have a broad range of applications and include technologies for genomic sequencing (genomics), mRNA quantitation and sequencing (transcriptomics), and protein (proteomics) or metabolite (metabolomics) identification and quantitation. In the area of genomics, next-generation sequencing (NGS)-based technologies have proven to be fast, accurate, and cost-effective and have revolutionized genomics research, healthcare, and medical practice. The rate of progress in this field is remarkable and promises to unravel a complete list of critical genes that are causative of diseases like cancer. These developments have helped advance personalized medicine, and the number of NGS platforms/instruments and approaches is constantly growing [4–7].

There are a wide variety of applications of NGS, including variant detection, whole-genome sequencing, whole-exome sequencing, and single-gene and multigene panels. NGS-based diagnostic tools are recent and their use in a clinical setting requires careful consideration of issues such as which tests to order, which vendors to use, how to interpret the results, and how to communicate results to patients and their families. For these reasons, it is necessary to understand the numerous applications, strengths, and limitations of the NGS-based diagnostic devices and approaches. Especially important is an appreciation of the limitations of NGS sequencing systems, including (1) substantial declines in performance for some regions of the genome (e.g., repeat regions, copy number variations and other variants, high-sequence homology, etc.), (2) informatics and workflow challenges, (3) identification of the appropriate set of tools needed, and (4) establishing which results are clinically actionable versus of unknown significance.

Mass spectrometry-based proteomics approaches provide an important class of massively multiplexed assays that have emerged as powerful methods for the identification, quantitation, and characterization of the proteins in a tissue or organism. A key application is in monitoring the disease or health status of a patient by providing comprehensive information on affected biochemical pathways and their protein products. Rapid advancements in techniques and protocols promise to make this technology a critical tool for identifying protein-based disease biomarkers, including cancer biomarkers for those under therapeutic interventions [8–10]. In addition, metabolomics is increasingly recognized as another powerful tool for monitoring the health status of patients by providing comprehensive data on metabolites and associated biochemical pathways [11,12]. These proteomics and metabolomics high-throughput approaches will have several analogous regulatory and clinical challenges with identifying low abundance molecules, difficulty of correlating profiles with disease status, lack of reference materials, analytic materials, and gold standards, and challenges with the bioinformatics pipelines.

## Regulatory Considerations

Precision medicine using omics technologies and approaches presents many unique challenges to drug and medical device regulation, and the FDA has referenced this in more than one of their regulatory science priority areas within their strategic plan [1]. To address these challenges, FDA has developed multiple guidance documents and concept papers, and has held US workshops and the global summits on regulatory science to address emerging regulatory issues [13,14]. Numerous precision medicines and corresponding companion diagnostic devices have been approved for marketing by the FDA [15]; however, most of these drug/diagnostic pairs involve a single biomarker. NGS and other omic technologies that interrogate multiple biomarkers or analytes in the same test are revolutionizing clinical management; however, they present additional challenges for the successful co-development of therapeutic product/diagnostic device pairs.

A recent breakthrough in the field saw the FDA approval of several NGS-based tests [16–19]. The Oncomine Dx target test is the first FDA-approved, NGS-based in vitro diagnostic for nonsmall-cell lung cancer designed to simultaneously screen patient tumor samples for 23 genes associated with nonsmall-cell lung cancer. Results from 3 of these genes can now be used to assess disease management options. The intended use and the summary of safety and effectiveness data of the approved in vitro diagnostics [20] describe the analytical and clinical validation requirements for FDA approval of these devices.

NGS-based assays involve complex multistep workflows that are subject to multiple (often poorly characterized) sources of variability. This presents numerous challenges to establish methodologies used by regulatory agencies attempting to establish the safety and effectiveness of an NGS-based test, while ensuring that the validation requirements are least burdensome. Additional challenges include ensuring truthful labeling when these assays can detect thousands of variants simultaneously with potentially ambiguous and nonuniform accuracy and clinical relevance. As will be discussed further later, there are additional complexities when such data are to be included in drug labeling. Therefore, it is critical to demonstrate the accuracy of NGS tests to enable them to generate data that can inform clinical decision-making. Regulatory agencies such as the FDA have organized several public workshops [21,22] to engage with stakeholders in industry, diagnostic laboratories, academia, and patient and professional societies to develop standards and tailored regulatory approaches to advance the oversight of NGS-based tests.

After engagement with the stakeholders mentioned earlier, FDA initially developed draft guidance and recently issued final guidance documents that provide recommendations for development and validation of NGS-based tests to aid in the diagnosis of germline diseases and conditions. These include the analytical standards guidance [23] and the database guidance [24]. FDA has also created precisionFDA [25], a cloud-based portal, that engages a community of over 2500 users across the world to collaborate and help define standards for evaluating NGS tests including analytical pipelines. It is a community-based research and development portal for testing, piloting, sharing data and tools, and validating existing and new bioinformatics approaches to NGS processing.

As we consider proteomics and metabolomics approaches, and the integration of these with genomic data, the need for validated reference materials, analytic materials, and bioinformatics tools and methods will all be important considerations for the development and use of emerging approaches.

## Key Areas and Recommendations

**Fig. 1:**
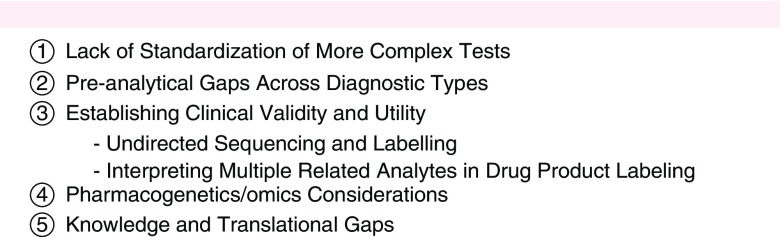
Key regulatory science gaps. These 5 key areas were identified as regulatory science gaps, with findings and recommendations provided for each to help advance precision medicine.

The Forum identified several regulatory science gaps, and the following provides findings and recommendations to help advance precision medicine (see Fig. 1).

### Lack of Standardization of More Complex Tests

Regulatory science governing complex biochemical tests is a relatively new area for regulatory agencies to consider. Several regulatory science gaps exist that impede the prediction of product safety and efficacy, specifically for standardizing assays that aim to measure large protein measurands (targets), analyze NGS genomic data, or quantify cell-based assays (whether in formalin-fixed paraffin embedded or frozen tissue), groups of covarying metabolites, or DNA/RNA targets. Regulatory science does not yet have “best practice guidelines” for complex biochemical tests. One major obstacle to such a guideline is the observed variability among specific attributes of measurands for predicting clinical outcome. This includes determining what proteoforms of a protein measurand are clinically relevant, and how different assay platforms can differentiate between these proteoforms. As a consequence of these gaps, there is a lack of understanding of the specific relationship between preclinical tests and patient response. Looking to the future of increased data and the use of predictive analytics, regulations, and/or guidance documents will need to be developed to set standards for NGS, metabolomics, and proteomic testing in addition to addressing the observed discordance among different platforms and laboratories. Lastly, it will be important to consider how regulatory science can normalize approaches for applying statistical tools appropriately to complex clinical assays.

There are significant barriers for achieving standardized clinical testing for complex assays, particularly as well-characterized certified reference materials and reference data do not yet exist. This is primarily because of the extraordinary analytical challenges associated with producing stable, homogenous, well-characterized, matrix-matched, exact-matched materials that are commutable or interchangeable for variable platforms, to our ability to properly define clinically relevant measurands, and to the difficulties with reproducing population heterogeneity in standards or reference materials development. These challenges understate the concern of whether standards can be functionally correlated well enough to satisfy regulatory requirements.

#### Recommendations

Although the challenges are great, there are many groups working together to address these barriers. Domestic (e.g., FDA, National Institute of Standards and Technology, United States Pharmacopeia) and international organizations (e.g., International Federation of Clinical Chemistry and Laboratory Medicine, Clinical & Laboratory Standards Institute, International Organization for Standardization-ISO), along with academia and industry should begin to identify potential assay biases and suggest changes. These groups must work closely with each other to precisely define the clinically relevant measurand structures necessary to target, and the level of assay accuracy and precision required for regulatory purposes. All key players should contribute toward developing well-validated standards, reference materials, reference data, and statistical tools in various forms (e.g., cells, DNA/RNA, protein complexes, and tissue) for all potential variations that meet clinical relevance. Finally, these materials, data, and knowledge should be made publicly available, both domestically and internationally to be scrutinized and improved upon by clinical and regulatory communities.

### Preanalytical Gaps Across Diagnostic Types

The development of safe and effective diagnostic tests that use the analysis of genomic, proteomic, or metabolomic data depends on the initial selection, collection, and storage of relevant patient samples into a suitable biobank or other resource. Several barriers currently exist in this preanalytic space that impact a broad range of omics-based diagnostic tests. Although the importance of biorepositories and biobanking has been an area of significant focus, concerns regarding reliability, accuracy, and completeness of samples and critical metadata continue to present challenges. This includes concerns regarding sample heterogeneity, assurance of sample quality, and confidence and completeness of linked metadata.

#### Recommendations

A consistent and clear approach to select and collect samples and associated metadata is required. Similar to the approach taken for chain of custody for evidence and Good Laboratory Practice quality standards, a well annotated data trail is essential to further ensure confidence in the quality and reliability of these samples while using an automated and transparent process. Although a completely harmonized process for all biorepositories may not be feasible, a well-documented process for sample collection, processing, analysis, preservation, and quality control procedures will be a critical step to limit variability and ultimately advance precision medicine through the development of improved diagnostics and therapeutics. The increased adoption of blockchain and alternate approaches to reliably and securely record and share data provides one approach to consider to address these requirements [26]. This will involve partnerships between federal agencies (e.g., NIH, NIST, FDA), organizations (e.g., ISO, College of American Pathologists), and a broad set of institutions across a range of disciplines, from genomics, metabolomics, and proteomics, among others.

### Establishing Clinical Validity and Utility

Currently, the lack of standards and reference measurement procedures for determining clinical validity and utility of biomarkers leads to variable interpretation of results across labs and healthcare centers. Consistent evidence-based classification of biomarkers is important in the application of precision medicine. This is illustrated in cases where the presence/absence or type of a specific molecular alteration can result in diagnosis of a genetic disease or determining eligibility of a patient to receive a specific therapeutic product (e.g., specific variants for ivacaftor [27]). However, since molecular alterations often occur infrequently within a disease, they may not be adequately considered in clinical trials of diagnostic devices or therapeutic products. Therefore, well-defined methods are needed for classifying molecular alterations (e.g., pathogenic, deleterious, etc.) and subsequently establishing clinical validity/utility of the test [28–30].

Several barriers for establishing clinical validity and utility of biomarkers can be identified. These include a lack of accepted standards and reference measurement procedures, limited reliable data sources outside of the traditional controlled clinical trial paradigm (where patients with biomarkers that occur at low frequencies are often not well-represented), and the inherent complexity of most diseases where multiple underlying genetic alterations exist and often impact disease pathogenesis or response to therapy. Moreover, the definition of phenotype, particularly when based on current clinical practice, is impacted by quality of diagnostic testing, clinical experience, and decision processes and can impact the classification of associated genomic variants.

#### Recommendations

Several potential pathways were considered to mitigate these roadblocks and establish acceptable standards for demonstrating clinical validity and utility: (1) developing robust nonclinical methods (e.g., in silico, in vitro, ex vivo) that help inform pathogenicity of molecular alterations (or responsiveness to a drug or class of drugs) and (2) establishing methods to extract informative data from healthcare systems that may support clinical validity/utility of tests (or responsiveness to a drug or class of drugs). Additional recommendations include requiring enhanced transparency of the data supporting the evaluation of clinical validity and utility. Important data elements that should be made publicly available (including for clinician use) include (but not limited to) study references, analysis of inclusion/exclusion criteria used in supportive studies, method of measurement used, and existing diagnostic criteria/guidelines (at least referenced) active at the time of phenotype/disease.

### Pharmacogenetics/Omics Considerations

#### Undirected Sequencing and Labeling

Although targeted sequencing approaches that focus on specific genes or mutations remain the primary approach for many current diagnostics, the use of undirected sequencing using whole-genome sequencing and whole-exome sequencing will continue to proliferate. How variants uncovered by undirected sequencing will be reflected in drug labeling in order to inform drug utilization remains an unsettled question [31]. As mentioned earlier, undirected NGS sequencing is uncovering large numbers of variants, and for many variants the manner in which they should affect drug utilization and selection, and how this should be reflected in drug labeling, will be imperfectly understood.

#### Recommendations

In the long term, in order to incorporate pharmacogenetics/omics concepts, one approach would be to have 3 classes of “claims” considered and reflected in the eventually constructed curated variants database: (1) the first level of claim would note that the meaning of a given variant is not currently known, (2) the second level of claim would note that the variant may be involved in a disease process or prognosis, and (3) the third level of claim would note that the variant may be able to inform a treatment course, which could include pharmacological and nonpharmacological treatment. If information on a given variant were found valid to support drug utilization or selection, then it could be included in approved drug labeling.

#### Complexity of Simultaneously Interpreting Multiple-Related Analytes in Drug Product Labeling

There are examples of multigene assays, such as a MammaPrint to inform chemotherapy decisions for women with breast cancer, that have received FDA approval with a specific claim [32]. However, complex multianalyte tests, such as proteomic tests and transcriptomic tests, are becoming more common and will increasingly require modified regulatory science paradigms, particularly as these approaches are integrated. An illustrative example in this area is the characterization of what is called the “interferon signature” in various highly complex autoimmune diseases such as lupus [33]. Historically, biologically relevant biomarkers have involved a single analyte, and regulatory practice has been to convert the single marker from a continuous variable to a discontinuous variable through assignment of a scientifically informed “cut-point” or multiple categorical assignments. This practice tends to simplify application of the biomarker, but this simplistic paradigm is not practical nor scientifically supportable when a complex network of often-related and correlated analytes is investigated and interpreted. This presents an analogy to the challenges associated with in vitro diagnostic multivariate index assays, which combine the values of multiple variables and provides a patient-specific result intended for use in the diagnosis of disease or in the treatment or prevention of disease (but the derivation of the result is often challenging to verify) [34].

#### Recommendations

New concepts will need to be developed to address the richness of multianalyte methods, perhaps through something analogous to a composite clinical endpoint in which each analyte is weighted based on its predictive ability. It will also be necessary for regulatory science paradigms to be devised which recognize that correlation with such a complex biomarker profile is more likely to form a gradient (of disease activity, or drug responsiveness) rather than a binary situation as is often reflected in current labeling.

In addition, a framework for continuous updating of product labeling to address complex profiles and to use rapidly expanding sources of clinically relevant information should be developed. This recognizes both the complexity of clinically relevant relationships, and the emerging practice of omics information being presented to clinicians through software decision tools that incorporate individual patient data in sophisticated modeling technologies [35,36]. One question is whether such models themselves may, in the future, provide and directly update actionable labeling information, particularly at the small group or individual level. This raises a number of potential questions for implementation and use, including the validation and optimization of underlying predictive models (for relevant background see [37]).

### Knowledge and Translational Gaps

Issues surrounding education and training in the omics domain may be similar to other innovative technologies used in a clinical setting, and thus may not raise unique concerns [38]. However, there may be complexities in the clinical setting that affect risk and appropriate clinical decision-making. Challenges such as the interpretation of treatment options provided through software, clinician attributes (e.g., training, experience) and interaction with patients, cultural influences when interpreting medical information, and cost-effectiveness assessment of omics technologies versus other treatment options have not been fully assessed.

For example, clinicians may not fully appreciate the complex and probabilistic relationships involved in the assessment of a particular patient’s treatment options, including consideration of potentially less expensive alternative (nonomics) approaches. Patients may inaccurately believe that their “genes” will directly tell them, in a deterministic way, if they will get a disease or be cured. Such considerations may inform the content and presentation of information.

#### Recommendations

This area should be recognized as an integral part of omics technology implementation, using a multidisciplinary approach that includes disciplines of psychology, economics, sociology, and anthropology. As part of a broad framework to address knowledge requirements in the application of precision medicine, the existing regulatory science competencies [39] could be further updated to guide training in this area.

## Conclusions

The areas outlined earlier provide a roadmap to help address some of the most critical regulatory science gaps to advance the approval and adoption of omics approaches for precision medicine. Although challenges lie ahead, there is significant potential for these technologies to transform patient diagnosis and therapeutic interventions based on personalized molecular profiles. As with the Regulatory Science to Advance Precision Medicine Forum itself, progress in this area will require partnerships among government, industry, academia, foundations, and other stakeholders.

## Supplementary material

For supplementary material accompanying this paper visit https://doi.org/10.1017/cts.2018.330. click here to view supplementary material
