# Supplementary material for: The integration of emerging omics approaches to advance precision medicine: How can regulatory science help?
Source: J Clin Transl Sci. 2018 Dec 6;2(5):295–300. doi: 10.1017/cts.2018.330 (PMC6390395; doi:10.1017/cts.2018.330)
Supplement: Supplementary file 1 [file S2059866118003308sup001.docx]

**Regulatory Science to Advance Precision Medicine Forum**

**A Meeting by University of Rochester, PhRMA Foundation, and University of Virginia**

**Wednesday, September 27, 2017**

**9:00am – 3:30pm**

**PhRMA Foundation**

**950 F Street NW**

**Washington, DC 20004**

**Agenda**

9:00 am Continental Breakfast

9:30 am Welcome *(Eileen Cannon and Bill Chin, PhRMA Foundation)*

9:35 am Review of Meeting Objectives *(Joan Adamo and Scott Steele, University of Rochester)*

9:45 am 3D Printing of Medical Products: Presentation(s) and discussion

- Emerging Science *(John Fisher, University of Maryland)*
- Considerations to Inform Regulatory Decision-Making *(Richard McFarland, ARMI)*

10:45 am Omics for Precision Medicine: Presentation(s) and discussion

- Emerging Science *(Shashikant Kulkarni, Baylor College of Medicine)*
- Considerations to Inform Regulatory Decision-Making *(Michael Pacanowski, Soma Ghosh and Zivana Tezak, CDER / CDRH FDA)*

11:45 am Discussion and Charge to the Breakout Groups *(Scott Steele, University of Rochester)*

12:00 pm Working Lunch: Breakout Group Sessions

1:45 pm Break

2:00 pm Reports from the Breakout Groups and Discussion

- 3D Printing
- Omics for Precision Medicine

3:00 pm Review Progress and Identify Next Steps *(Robert Meyer, University of Virginia)*

3:30 pm Adjourn


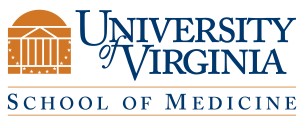

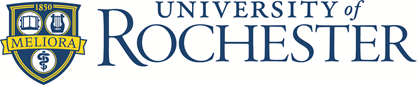

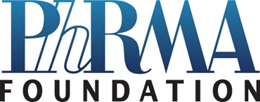


**Regulatory Science to Advance Precision Medicine Forum Participants:**

Joan Adamo, Director, Regulatory Support Services and Assistant Professor, Biomedical Engineering, Clinical and Translational Science Institute, University of Rochester Medical Center

Robert Bienvenu II, Adjunct Assistant Professor, Daniel K. Inouye Graduate School of Nursing,

Uniformed Services University *(Currently with the University of Maryland, College Park, Maryland)*

Khaled Bouri, Office of the Chief Scientist, FDA

Jennifer Brown, Director, Clinical Research Quality, Spectrum, Stanford Center for Clinical & Translational Research & Education

Bruce Burnett, Director of Regulatory Affairs and Quality, Duke Translational Medicine Institute, Duke University; Office of Research Support and Compliance, Office of the Director, NIH

Eileen Cannon, President, PhRMA Foundation

Christine Colvis, Director, Drug Development Partnership Programs, National Center for Advancing Translational Sciences, NIH

Owen Fields, Vice President, Inflammation and Immunology – Regulatory, Worldwide Safety & Regulatory, Pfizer Inc.

John Fisher, Director, Center for Engineering Complex Tissues and Chair, Fischell Department of Biomedical Engineering, University of Maryland

Soma Ghosh, Office of In Vitro Diagnostics and Radiological Health, CDRH, FDA

John Gigantino, Director, Global Regulatory Affairs, Sanofi

Mathangi Gopalakrishnan, Research Assistant Professor, Center for Translational Medicine, University of Maryland

Warren Grayson, Associate Professor, Department of Biomedical Engineering, Johns Hopkins University

Heather Hatcher, Clinical and Translational Science Institute, Wake Forest Baptist Medical Center

Scott Hollister, Professor, Wallace H. Coulter Department of Biomedical Engineering, Georgia Institute of Technology and Emory University

Rosemarie Hunziker, Tissue Engineering/Regenerative Medicine Program Director, National Institute of Biomedical Imaging and Bioengineering, National Institutes of Health

Christina Jones, Research Chemist, Organic Chemical Measurement Science Group, NIST

Karl Kieburtz, Professor, Neurology, University of Rochester Medical Center

Shashikant Kulkarni, Professor & Vice Chairman for Research, Department of Molecular and Human Genetics, Baylor College of Medicine; Chief Scientific Officer and Senior Vice President, Baylor Genetics

Michael Liebman, Founder, IPQ Analytics, Adjunct Professor of Pharmacology and Physiology,

Drexel College of Medicine

Sheng Lin-Gibson, Leader, Biomaterials Group, Biosystems and Biomaterials Division, NIST

Mark Lowenthal, Research Chemist, Bioanalytical Science Group, NIST

Don McClain, Director, Clinical and Translational Science Institute and Professor of Medicine, Endocrinology and Metabolism, Wake Forest Baptist Medical Center

Richard McFarland, Chief Regulatory Officer, Advanced Regenerative Manufacturing Institute/ BioFabUSA

Sean Mellican, Takeda Pharmaceuticals, Inc.

Robert Meyer, Director, Virginia Center for Translational and Regulatory Sciences and Associate Professor, Public Health Sciences, University of Virginia

Andrika Morant, Clinical Translational Science Center, Weill Cornell Medicine

Michael Pacanowski, Office of Clinical Pharmacology, CDER, FDA

Daryl Pritchard, Vice President, Science Policy, Personalized Medicine Coalition

Henry Rodriguez, Director, Office of Cancer Clinical Proteomics Research, National Cancer Institute, NIH

Kelley Rogers, Federal Technical Program Manager, NIIMBL, NIST

Robert Schuck, Clinical Pharmacologist, Genomics and Targeted Therapy, Office of Clinical Pharmacology, Office of Translational Science, CDER, FDA

Noel Southall, Leader, Informatics, National Center for Advancing Translational Sciences, NIH

Scott Steele, Director, Regulatory Science Programs and Associate Professor, Public Health Sciences, Clinical and Translational Science Institute, University of Rochester Medical Center

Zivana Tezak, Office of In Vitro Diagnostics and Radiological Health, CDRH, FDA

Bruce Wilcox, SVP, Research & Development, Applied Proteomics, Inc.

Jeanne Wright, Clinical Research Project Manager, Michigan Institute for Clinical and Health Research, University of Michigan at Ann Arbor

Carolyn Yong, Team Leader, Device and Combination Products, Cell Therapies Branch, Office of Tissues and Advanced Therapies, CBER, FDA

Yuan-Shan Zhu, Clinical and Translational Science Center & Weill Department of Medicine, Weill Cornell Medicine


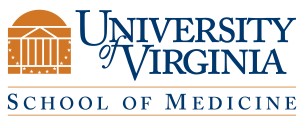

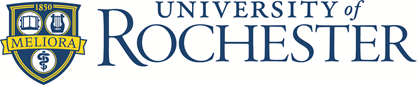

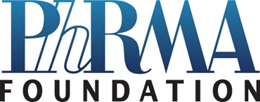


*The project is supported by the University of Rochester CTSA award number UL1 TR002001 from the National Center for Advancing Translational Sciences of the National Institutes of Health. The content does not necessarily represent the official views of the National Institutes of Health.*
